# Debtor and Creditor

**Published:** 1879-04

**Authors:** 


### DEBTOR AND CREDITOR.

"How I wish father was here now," and Mrs. Smith looked complacently at the pan of cream-biscuits she had just drawn from the oven. "I've had such good luck with these, and he's so fond of them, too. Run, Jimmie; run down to the gate, child, and see if he isn't coming. I do hate, of all things, to have cream-biscuit wait, and these are *so* nice;" and she turned them from the dripping-pan on to a side-table, and broke them up.

They did indeed look tempting, so light and white, with such a delicate shade of amber-brown on their crusts. When the last one was piled on the plate, a most appetizing odor diffused itself over the old farm-kitchen—an odor that would have made a dyspeptic sigh as he broke in halves his hard brown crackers. Covering them with a towel, fresh from the drawer, she set them on the tea-table, and then resting a hand on each hip, surveyed it carefully, to see if all was there.

It was a genuine old-fashioned Yankee tea-table, such a one as makes our mouth water only to remember, with a homespun linen cloth, snowy as drifted flakes, and yet in its Sunday creases; with a quaint mulberry-colored tea-set; tiny silver spoons, that had grown thin with the handling of three generations, and horn-handled knives and forks, scoured to a mirror-brightness. Cream from the morning milk floated in the little pitcher; pure maple-sugar filled the bowl; a pat of butter, golden as the wheat-sheaf that was stamped upon it, was flanked on the one side by a ball of new Dutch-cheese, and on the other by a plate of pickles, green and crisp as though fresh from the vines; a quart-bowl, just beside the biscuits, held circular slices of beets, tinging the vinegar with the crimson-purple of claret wine; opposite was another, with cider apple-sauce, each great mellow quarter, mellow to the heart, yet perfect in shape, while the four corners of the table bore proudly the pies and cakes; pumpkin-pie, ruddy as the old brick-oven in which it had been baked; apple-pie, with upper-crust that dropped into flakes as you cut it; cookies, with caraway-seeds in them for flavoring; and doughnuts, brown as a berry on the outside, and creamy-white in their centers.

"Yes, I believe I've got all; now, if he would only come!" and she turned to the fire-place, and lifted the tea-kettle from the hook, and set it on a warm corner of the ample hearth.

"He's coming, mother; he's 'most here;" and nearly out of breath, Jimmy bounded into the kitchen, "and I guess he's got the money too, for he looks ever so glad. Won't you be glad, too, mother?"

"Yes, indeed, child, the dear knows I will; but run, now, and wash your face and hands, and call Susan to set the chairs up. I'll make the tea in a hurry."

"Supper all ready! Well, I'm glad of it; for I tell you what, mother, I'm hungry as a bear;" and the broad-chested, sturdy, sunburnt, yet genial-looking farmer, drew off his overcoat, and pulled off his cap, and handed them to his wife, and then run his fingers back and forth through the blaze that went up the chimney, rubbing them briskly the while.

"It's chilly riding, and I shouldn't wonder if we had a frost to-night. Did the children gather in all the pumpkins to-day?"

"Pretty much, father. All that's fit to cook ——"

"Some great bouncers, too," interrupted Jimmie;" it was all Sue and I could do to roll them."

"You'd better throw some old blankets over them to-night, mother. I don't want 'em brought in, as long as I can help it, for every day's sunning helps sweeten 'em. My old mother used to say it saved half the molasses to let 'em sun a fortnight."

"And so it does, father, but come, sit down now."

"Don't look much like hard times here, mother;" and Mr. Smith set down the cup of fragrant tea his wife had handed him, broke open the biscuit he had helped himself to, and spread the halves with a generous allowance of butter "Not much like hard times;" and he deposited a brimming spoonful of apple-sauce on his plate, and dipped his fork into the bowl of beets. "We've thought we knew something about 'em; but I tell you, mother, we've got to fare slimmer than this before we feel 'em to speak of. If you just could only have set down to the table I did to-day noon, I reckon—well, I reckon you'd a-choked up, mother. You see, I met cousin Sam Jones in the street, just as I was going down to the tavern to get a dinner, and nothing would do, but I must go home with him. I didn't want to a bit, for I knew they must be short about these times; but he wouldn't take no for an answer, and so I went. I was sorry enough, though, when I saw Sary Ann, for she looked so flustrated; but she shook hands with me as warm as ever, and said she was glad to see me, though if she'd known I was coming, she'd a-tried and tossed up something a little better. Well, we set down to the table; but dear me, mother, I could have eat every mouthful of it myself, and then had room for a decent dinner."

"What *did* they have, father?"

"Have, mother; why, they had a piece of steak, just about as big as my hand. I don't believe there was over a pound and a half of it, and about a dozen little crazy potatoes, not one of them a bit larger than my thumb, a slice of butter about as thick as one of those cookies, and just about as big round, and a small loaf of baker's bread, that had about as much substance to it as a soap-bubble, and a pitcher of water."

"No pie or pudding, father?"

"Not the first mouthful, mother. I tell you, I didn't eat much; told 'em I wasn't very hungry, for I'd been lunching on doughnuts all along the road. 'O dear!' says little Moll, 'I wish we could have doughnuts. We haven't had any for ever so long. Why don't you make some, ma?' Sally Ann, she colored up, and says, kindly, softly: 'Hush, Molly; you know the times are too hard for father to buy lard to bile 'em in.' Well, that kinder started 'em; and sich a story as they had to tell, mother! Dear me, but it made my heart ache only to hear it. His wages have been cut down half, and he can't always get that when it is due, and sometimes they don't for days have any thing to eat but hasty-pudding and molasses, and sometimes they even have to go without the molasses. Sally Ann said she hadn't had a bit of tea or sugar for two months, nor an egg, nor a pie or a cake. I'll tell you what I did: I just went right down to the wagon, and got that basket of doughnuts—I hadn't eat 'em half up—and carried 'em to the children. Mercy, but how they did pounce on 'em! I couldn't think of any thing but a half-starved cat coming across a stray mouse, they grabbed 'em so. And such a shout as they gave when they saw the slice of cheese! Sally Ann said it was more than a year since she had tasted a bit."

"Dear, but how funny—not to have cheese in the house all the time. Why, I reckon we've got forty now, up in the cheese-room."

"Twenty, Jimmie, twenty; don't you stretch things so. I do wish I'd known it, father, before you started. I'd sent her one, and a roll of butter, and a load of vegetables. You might have carried 'em just as well as not, if we'd only thought of it; but I never supposed folks—decent kind of folks, I mean, such as they are—ever had to do without such things."

"Nor I either, mother, and it set me to thinking, as I was coming home; and I believe you and I have often done rich people wrong, when we've called 'em stingy because they didn't divide with the poor around 'em. I don't believe it's stinginess a quarter of the time. It's because they don't think. They've so much of every thing themselves, they don't realize how others do live. Sally Ann and Sam might have thought we were stingy to-day, because I didn't bring 'em in a load of one thing and another from our farm; I say they might, if they didn't know just what we really are. But you and I know that wasn't the reason. But never mind; I shall go down again in two or three weeks, and I reckon I'll make the springs bend some with the load I'll carry 'em then. I'll put in a good lot of potatoes and turnips, and sich small trash, and half a dozen good-sized pumpkins, and four or five bushels of apples, and——"

"And I'll send him some of my nuts," cried Jimmie, "a great bag of nuts, all mixed up, walnuts and butternuts and chestnuts. I reckon they'll make little Moll open her eyes."

"And I'll send cousin Sally two of my chickens;" and little Sue's eyes sparkled, and dimples danced all over her sunny face.

"And I'll put in a roll of butter and a cheese and a ten-quart pail of apple-sauce, and I'll bake one of my biggest loaves of bread for her; I reckon home-made bread, wet up with new milk, will be quite a treat to them."

"And mother, put in some doughnuts and cookies," cried Jimmie.

"And O mother! make her a nice loaf-cake, with raisins in it and sugar on the top—white sugar, I mean."

"Yes, yes, Susan, and I'll send her a couple of gallons of new milk, and a quart or so of sour cream, to mix up a few biscuits. I don't suppose she's had a cream-biscuit these two years. Dear me; but I don't know how city folks do live so from hand to mouth. I reckon Sally Ann is sorry enough now she ever persuaded Sam to go there to live. To be sure, he wasn't making much at his trade here, but then their rent was only a trifle; and their garden kept them in vegetables the year round, and they had a cow and could make their own butter, and once in a while change milk with a neighbor, and make a cheese or two, and they could fatten a couple of pigs every year, and keep hens and have fresh eggs, and raise all the fruit they needed but winter apples. Their currant-bushes were doing so when they left, while their cherry-trees almost broke down, and their plums and peaches would have borne in a year or two, a plenty. And now, they don't have any thing but what they buy. It's too bad. I wouldn't stand it."

"Nor I either, mother. This putting one's hand in his pocket, every time he wants a bite, an't just the thing, according to my notions. Sam tried hard to have me go when he did. But I gave him a right flat *no*. Says I, Sam, may be I won't make as much money as you, but I'll live a deal sight better. Poor fellow, I wonder how he'd feel to happen in jist now, and set his eyes on this table. And yet we don't think this is any thing extra; at least nothing but the biscuits." And swallowing the last bit of the fifth one, he reached out his plate for a piece of the pumpkin pie.

Mrs. Smith thought it a favorable opportunity to ask the question that had been on her lips ever since he came in.

"Did you get your money, father, to-day?"

"I reckon I did, mother," and he clapped his right hand on his breast-pocket. "I reckon I've got a hundred dollars hid here; bran new bills, too, every one of 'em. No, not quite a hundred; for after I got 'em, I went and bought a pound of tea and a dollar's worth of sugar, and gave 'em to Sally Ann, for I couldn't bear that any of my connections, and a woman, too, should be drinking cold water all the time. Don't they look good?" and opening the old leather pocket-book, he took them out and counted them over. "Five tens is fifty; nine fives is forty-five, and this three is ninety-eight; just it."

"I'm so glad you got it, father. I've worried all day for fear they'd disappoint you, and goodness knows what would have become of us this winter, if they had."

"And I'm glad, too," shouted Jimmie, "for now I shall have new boots and a new cap, a store cap, such as other boys wear, and a new overcoat out of father's old one, and a new jacket and pants. Hurrah, boys, an't I glad?" and he shoved his chair back hastily, and picking up the old cap which his mother had fabricated the winter before, out of bits from her bundle-bag, he sent it, as he said, "a-kiting."

"And I'll have a new dress, won't I, mother?" said little Susan very earnestly; "a new delaine dress—a red one, with little black dots over it. O dear, won't it be funny, to have a dress right out of the store. I've had to have mother's old ones cut over for me, till I'm tired. And I'll have your cloak now, won't I, and new shoes and a belt, mother; all the little girls wear belts ——"

"And what'll this little fellow have?" said the mother, cheerily, as she took up the crowing baby out of the cradle. "He'll have a new dress, too, won't he, father?" and she held the little soft face close to the farmer's lips.

"May be, may be," he said, as he tossed the little one to the ceiling half a dozen times. "There, take him, now, mother, for I must unhitch the horses and get them into the stable. *Bonnies* are tired and hungry by this time," and he hurried away.

The chores were all done and the children put to bed. The farmer sat in his easy-chair, which was tilted back against the oven-door, and looked the picture of homely comfort, with his legs crossed so lazily, his arms folded so cosily, and his "pipe of clay" set so snugly between his lips. His wife sat in her low rocker, with the stand drawn closely to her, though the blaze from the hickory fire rendered the light of the candle almost unnecessary. Her knitting-work lay beside the snuffers ready to take up, as soon as the last stitch was set in the long patches with which she was covering the holes in the knees of Jimmie's trowsers. The cradle stood near by, so close that her foot touched it lightly if the baby stirred.

The fire crackled and blazed; the farmer smoked and seemed lost in thought; the farmer's wife sewed, and she too seemed lost in thought.

By and by, she hung the mended trowsers on the foot of the cradle, saying as she did so: "There, I hope it's the last time I'll have them to patch." Then she took up the double mitten she was knitting for her husband, and her fingers flew as though his hands were bare, though if the truth be told, he had two pairs yet in the stocking-bag, besides those he yet carried in his pockets. But she was a thrifty wife, and always ahead with her knitting.

"I'm so glad you got that money, father," she said after a while. He did not answer her, but puffed away at the old pipe.

Presently she spoke again. "Shall you be using the team to-morrow, father?"

"Why, mother?"

"Because, I thought if you wasn't, I'd have you drive me up to the store. Now we've got the money, we may as well get our clothes first as last, and have them cut, and then when I get a minute's time I can be making them. It'll take me nearly all winter, any way. I don't suppose Grey's got his winter stock yet, but we can buy twenty or thirty dollars' worth out of what he's on hand."

Mr. Smith did not reply at once. He smoked out his pipe, knocked out the ashes, and laid it on the shelf. He set his chair forward on its four legs, drew off his boots, and planted his feet on the front round, and then putting an elbow on each knee, he rested his face on his hands. It was his usual attitude when he was going to talk seriously, and his wife's heart began to rise in her throat.

"What would you have done, wife, supposing I hadn't got that money?" he said, after clearing his throat with sundry hems and haws.

"Why, I'd had to got along without it, I suppose," she answered, rather curtly, "but the dear knows how, though, for I've twisted and turned every which way the last year. We're every one of us nearly naked for clothes—every thing we've got is ready to drop to pieces. But what makes you ask such a question?"

"Because," speaking very slowly, "I've been thinking that if we could possibly make our old clothes do this winter yet, I'd take that money and use it for something else."

"But I thought you'd said, over and over again, that if you ever got that hundred dollars you'd spend every cent of it for clothes, and so get a good start again."

"So I have, mother, so I have, but—well, I'll just tell you what started me to thinking we'd perhaps better use it some other way. Just as I got off this morning, I found one of old Ned's shoes was loose, so I went round to the smith's to have it fixed. Well, it was pretty early, you know, and they hadn't much fire yet, and the shop was open and cold, so I thought I'd run into Johnson's and warm me a bit. They were jist sitting down to breakfast ——"

"How is *Miss* Johnson, father? I have never seen her since her baby was born, and it must be over three weeks old now. Dear me, how time flies! It's too bad, too, when I thought so much of her."

"She's poorly, mother—thin as a June shad and white as a ghost, and the puniest baby you ever set eyes on. But as I was saying, they was jist sitting down to breakfast, and what do you think they had—rye griddle-cakes and milk-gravy ——"

"No meat or potatoes?"

"Not the first mouthful, nor any coffee, nor any tea, but catnip ——"

"Catnip ——"

"Yes, mother, catnip. 'You've eat your breakfast, I reckon,' says he, as he drew up his chair. 'If he hadn't,' says she, 'he wouldn't relish ours much,' and then she turned her head away, but I saw her wipe her eyes with her apron."

"Poor thing! but, father, I always thought Johnson was a good provider."

"So he is, mother, so he is; but just wait till I tell you. 'It's hard fare,' says he, as he took up a cake; 'I didn't think once I could have stood it to have gone without meat or potatoes, or butter or coffee, for breakfast, but these hard times play the deuce with a fellow's earnings.' 'But I thought you was doing pretty well now,' says I. 'Well, so I am,' says he. 'I turn away work every week, but the trouble is, no one has any thing to pay with; it all goes on to the book.' Well, I tell you, mother, that made me feel rather uncomfortable, for I couldn't help thinking of the hundred dollars I owed him."

"Yes, but he agreed to wait a year when you spoke of getting the wagon made, you know, and he's got your note for it, and it's bringing him interest all the time."

"I know it, mother, but—well, when I got ready to go, he went out with me, and says he: 'I've been thinking about coming over to see about that note, neighbor Smith. I tell you, we're pretty hard up just now. We an't had a spoonful of tea or coffee, or a bit of meat or wheat-bread in the house, since the baby was a week old, and we have to let all the butter go that we make now, to pay old Granny Boone for taking care of Mary a fortnight. She ought to have had help a month, for she's mighty thin this fall, but we were too poor.' 'But can't you get trusted at the store?' says I. 'Yes, I can, but I owe Grey fifty dollars now, and I hate to ask him to let the bill run any longer, for I know he's in a tight place, too; and then I owe the butcher ten dollars and the doctor ten, and I signed ten for the minister, and I know they all want it. There's all the debts I owe in the world, and a hundred dollars would make me square, you see, and give me a little start, too, and I've been thinking if you could pay that note now, I'd throw off interest—yes, and ten dollars of the principle, for ninety now is worth more to me than a hundred and six will be next spring.' Well, I told him just how it was—how we'd calculated to put that into clothes and sich like, but he looked so sorrowful that I told him I'd speak to you about it, and if you thought we could get along till spring with the old clothes, why, I'd take up the note now. What do you say, mother?"

Mrs. Smith did not answer. She had dropped her mitten and was looking dreamily into the fire.

"We should be out of debt then, you know," said the farmer, after a while, "and that would be such a comfort. We've had a pretty hard tussle with the world, getting all these mortgages paid off. I reckon it'll be many a long day before the old farm gets saddled with another one, but father, though a mighty hard working man, was always too easy with folks; he never would say no."

Still his wife did not speak. She was thinking of the many, many things she had "lotted so" on buying with that hundred dollars.

The clock struck nine. "Bed-time," said the farmer, giving himself a good shake before the fire, "and I'm ready for it, too, for I'm about tired out. Here, mother," taking out his pocket-book and handing it to her, "you take care of this; it's mighty precious just now, and mind you, mother, do jist as you think best. If you've really set your heart on spending it for clothes, why, take it and buy 'em. But if, after thinking it all over, you find you can manage any way to make our old ones do, why—but do jist as you're a mind to. I don't care a copper, as far as I'm concerned, only I hate to think of *Miss* Johnson drinking catnip-tea and we owing her man."

"So do I, father."

Mrs. Smith's voice was husky as she spoke, and it was only after she had swallowed hard two or three times, that she was able to add: "I'll think it all over, father, before I go to sleep, and see what can be done."

She did so. Long after her husband's eyes were closed in sound slumber, she lay wide awake beside him, devising, calculating, and wondering. "If it wasn't for his and Jimmie's boots, and Susan's and my shoes, I do believe I could manage after all, but I can't make over boots and shoes. Well, well, I'll go to sleep, now — perhaps I can think of some way in the morning to get them. Poor Mary Johnson — drinking catnip-tea, and living on rye-cakes, and her baby only three weeks old, it isn't right," and then she said her prayers, oh! how fervently! and dropped off into a sleep, sweet and sound.

Her morning work was light the next day, for she had washed and scrubbed and baked and churned on Monday, that in case her husband got his money she could get an early start to the store. By the time the children were off to school and the baby had settled himself for his morning nap, she was at liberty to commence her rummaging. She went first to the "south room" —parlor, city folks would have called it, but she was country bred, and satisfied with the same quaint name her husband's mother had given it when the house was built. Opening the drawer of the bureau, she commenced taking out her husband's shirts and looking them over carefully. "Well, I declare," she said to herself, as she replaced them, "they an't worn so bad as I thought; if I put a new bosom and collars and wristbands on two, they'll be nearly as good as new, the muslin isn't worn any, hardly, and the other two will bear mending some time yet. The one he wore yesterday is whole, and pretty strong too; that's five, and the sixth he's never had on yet, for I've always made it a rule to keep one out of every set all new and nice, in case any thing should happen." She sighed as she spoke, for well she knew what that "any thing" meant.

"Yes, I guess I can keep them on another year, or till spring at any rate, for he don't often wear white shirts in winter, and it's so lucky I made up so much flannel last year. He didn't want me to, but I seemed possessed to do it. If I hadn't, I don't know what would have become of us now, with having to sell all our wool this summer to pay off that last mortgage. Thank fortune it's paid too, and the old place is safe." And then she took out the Sunday vest and neck-kerchief. "Well, they don't look so dreadful bad, after all; I guess if I iron this out nice, I can fold it so as to hide the old creases, and then it'll do almost as well as new, and I can put new buttons on the vest, and bind it over, and it will last quite a spell;" and she closed the bureau and went to the "north room," where behind a sheet hung her own and husband's best clothes. "They're pretty thin," examining the pants, "pretty thin, but then if I make him a new pair out of that piece of full cloth that was left over last winter, why, he won't need to wear these only on Sundays, and he can take them off as soon as he comes from meeting, and that'll save them a good deal," and she hung them up and took down the coat. "It's most threadbare in spots, I declare, but then he can wear his every-day one under his overcoat this winter, and if he keeps that buttoned up close, why, no one will be the wiser. I did hope, though, to have got him a new coat this fall, but — well, this must do some way. They cost so much," and she replaced it and took down his best overcoat. She shook her head as she examined it, but presently her eyes brightened, she had remembered that there were pieces enough left to put on a new collar and new cuffs, "and that, with new buttons and new binding, will make it look quite respectable. As for his cap, he must slip that into his pocket when he goes into meeting; it'll do well enough elsewhere. Now I must look at mine," and she spread out on the bed a purple merino dress, a cloak of brown circassian, and a black alpaca skirt and basque. "If they only hadn't been turned once—oh! I've just thought, I'll dye the merino and the cloak, dip the alpaca, and then, when I've made 'em all over nice, they'll do as well as new." And she hung them up, and took down a band-box. "I was in hopes to have had a new bonnet, and had this made over for Susan, but I guess it'll do this winter. Poor Sue, she'll be so disappointed about the red dress; she's lotted on it so much. There, I've just thought what I'll do; I'll take that delaine I've had for a good dress these three summers, and die it crimson. There'll be enough of it to make the baby one, too, and the cape'll make each of them a hood. Susan will think just as much of it, if it's only red, and I'll buy her a belt some time, when I have a little butter-money. And that linsey of mine, that I was going to take for a petticoat, will make her a good every-day dress, and hers that she wore last winter will do for the baby; so they're fixed out. No, there's her cloak. Oh! I'll dye her old one when I'm about my clothes, and that'll make it as good as new in her eyes. Now for Jimmie. Let me see; I do believe there'll be enough of that full cloth to make him a Sunday pair, and I'll cut over some of his father's old ones that I have been saving for a new carpet, for every day wear. And there's those old coats of his grandpa's, which I never could bear to think of ripping up, they'll make him all he needs this winter, if I cut them over; so he's fixed, all but his cap; but then caps are cheap now, and I'll save in groceries some way, and get him one at the store with my butter-money. Now if it wasn't for the boots and shoes. I do wish I had something to sell that would bring money enough to get them. And I have—I have—there's these feathers; I did once think I never would let them go, but I will — *I will!*" she repeated, emphasizing the word heartily, and then she went into the kitchen again, and began to fly round to get an early dinner.

"Going to use the team this afternoon, father?" she asked, as the farmer shoved back his chair from the table.

"No, I guess not; why?"

"Why, I'd like to go and sit with *Miss* Johnson, awhile, father — and, father, you may take up that note, while we're there. I've made up my mind to make what clothes we've got do, somehow or other. Here's the money," and she brought the pocket-book from the "south room." "I've put in two dollars that I'd saved up out of my butter money, and as for the interest, why, I reckon you can fill up the wagon with some things that'll be as good as cash to them. I've killed and dressed a pair of chickens, and will put up a loaf of fresh bread and a pie or two, and a pail of apple-sauce."

"That's right, mother; and I'll load up with apples and garden sass and such like, that they have to buy. I feel as if I could give away half I own, to think I'm going to be out of debt. You get ready as quick as you can and I'll harness up in a jiffy."

Let us follow that roll of bills, and see how many hearts were made glad by the self-sacrifice of one farmer's wife.

Mrs. Johnson lay on the bed, faint and weak, and if the truth be told, just ready to cry. She was actually suffering for the want of suitable food and drink. She never could bear rye, and her stomach fairly loathed it now, and she did miss her tea and coffee so much. She had never complained to her husband, but her pale, thin cheeks, as they sat down that day to dinner, had so touched his heart, he could not swallow a mouthful, hungry as he was.

A rap at the door. She hastened to rise and open it. "Why, *Miss* Smith, dear woman, how glad I am to see you!" and she shook her visitor cordially by the hand.

"I'll help myself, Mary. Go and lie down again; you don't look fit to sit up. I never knew how poorly you was till father told me last night, or I'd a-tried and got out to see you before. It's no one but father," as a second rap was heard at the door; "come right in."

"How are you, to-day, *Miss* Johnson? haven't gained much since yesterday; where's Johnson—in the shop?"

"I expect he is, Mr. Smith; sit down, and I'll send bubby after him. Run, Harry, run, and tell pa who's here."

Mr. Johnson stood beside his bench with a chisel in his hand; but not, as usual, busy as a bee. He had no heart to work; "Where was the use," he muttered, "in these hard times, when a poor fellow, let him earn ever so much, can't get a drawing of tea for his sick wife? What's that, Harry—Mr. Smith wants to see me? well, I'll come, right away, too. I do wonder if he's going to take up that note; if I thought he was — but pshaw! it isn't often a man pays a note till it's due, when times are easy. Ah! Smith, how are you to-day? and you, *Miss* Smith, pretty well, eh? and there's the baby, too — don't look much like our little rat, here. But, sit down, Smith, sit down."

"I haven't time, just now, Johnson. Have you got that note about you? Mother, here, has concluded she'll make our old clothes do awhile longer, and pay up our debts, and as she wanted to come and visit awhile with your wife, I thought I'd come along and have that little business of ours all straightened. There," counting out the money, "there's the face of the note, a hundred dollars, and the six months' interest is out in the wagon, if you'll take it that way; if not, you must wait till butchering time ——"

"Don't say a word about the interest, neighbor," and great tears rolled down Johnson's cheeks, while his wife sobbed aloud; "and you must take back this ten, too," handing him a bill.

"No, no, not a cent. You made the wagon on honor, and it's worth all I agreed to pay for it. Take it all;" then twisting the note in his fingers, and holding it in the fire till it was all of a blaze, "no man can look me in the face now, and say I owe him a copper. Hard times won't trouble me any longer, for I can raise enough on the old place to make a living, let prices be ever so low, now I am out of debt; but I guess I'll be traveling. Mother wants me to go up to the 'Squire's, and sell some feathers for her. Come on, Johnson, and get that interest out of my way."

"There, that bag's apples, and that's potatoes, and that's meal, and here's a lot of turnips, onions, and beets, and some of my big cabbages, and a pumpkin or two; them things there in that bucket and pail is some little notions mother fixed up for your wife; you see, she thought something away from home might relish. Got 'em all? well, I'll be off, then," and he whipped up his horses and was soon out of sight. Mr. Johnson carried in the interest, and then, stopping only to take off his apron and put on his coat, started off for the village.

"I'll go to the minister's first," he said to himself; "for ministers are always hard up—the dear knows how they do live in these times."

